# Induction Chemotherapy‐Related Covert Cardiac Remodeling in Pre‐Autologous Hematopoietic Stem Cell Transplantation for Multiple Myeloma: A Retrospective Observational Study

**DOI:** 10.1002/cam4.70329

**Published:** 2024-11-12

**Authors:** Chang Dai, Weidong Lin, Fangzhou Liu, Xin Chen, Yuhan Chen, Yu Jiang, Jiaojiao Bai, Yidong Lv, Jianhong Zheng, Hai Deng, Xin Du, Shulin Wu, Yumei Xue

**Affiliations:** ^1^ Department of Cardiology, Guangdong Provincial People's Hospital (Guangdong Academy of Medical Sciences) Southern Medical University Guangzhou Guangdong P.R. China; ^2^ Guangdong Cardiovascular Institute Guangdong Provincial People's Hospital (Guangdong Academy of Medical Sciences) Guangzhou Guangdong P.R. China; ^3^ Guangdong Provincial Key Laboratory of Clinical Pharmacology Guangdong Provincial People's Hospital (Guangdong Academy of Medical Sciences) Guangzhou Guangdong P.R. China; ^4^ Department of Haematology, Guangdong Provincial People's Hospital (Guangdong Academy of Medical Sciences) Southern Medical University Guangzhou Guangdong P.R. China

**Keywords:** arrhythmia, autologous stem cell transplantation, cardiovascular toxicity, induction chemotherapy, multiple myeloma

## Abstract

**Background:**

Autologous hematopoietic stem cell transplantation (ASCT) has emerged as a cornerstone in multiple myeloma (MM) management, offering the prospect of prolonged disease control. However, the induction chemotherapy drugs required prior to ASCT carry cardiovascular toxicity (CVT), potentially leading to a range of cardiovascular complications.

**Methods and Results:**

This retrospective observational study, conducted at Guangdong Provincial People's Hospital from January 2020 to December 2023, analyzed 47 of the initial 173 patients who met the criteria. The cohort, comprising 22 males (46.81%) and 25 females (53.19%), had a mean age of 55.68 ± 11.38 years. They underwent various induction chemotherapy regimens, receiving a median of 5 (4–6) cycles of the course over an average duration of 7.10 ± 2.46 months. Before ASCT treatment following induction chemotherapy, echocardiographic findings indicated reductions in left ventricular end‐systolic dimension, right atrial diameter, E‐wave velocity, E/e' ratio, and the E/A ratio. The latter altered from a pretreatment value greater than 1 to posttreatment less than 1, marking diastolic dysfunction emergence or aggravation in 51.06% of patients. The electrocardiographic data indicate a reduced heart rate and prolonged P‐wave duration and P‐R duration, with an increase in arrhythmia incidence to 19.15% following induction chemotherapy.

**Conclusion:**

Induction chemotherapy, administered prior to ASCT in patients with MM, can lead to the emergence or aggravation of cardiac diastolic dysfunction and increase the incidence of arrhythmic events. Therefore, it is crucial to emphasize the importance of balancing the benefits and risks of induction chemotherapy to maximize its efficacy while minimizing CVT.

## 1 Introduction

Multiple myeloma (MM), a type of blood cancer characterized by uncontrolled proliferation of plasma cells in the bone marrow, is effectively treated with autologous hematopoietic stem cell transplantation (ASCT), a pivotal therapeutic strategy for this disease. ASCT offers a potential for long‐term disease control in MM. It involves the infusion of healthy hematopoietic stem cells to replace the diseased bone marrow and restore normal blood cell production. The process of ASCT for MM typically involves several stages, with pre‐transplant chemotherapy being a crucial component. This therapy, known as induction chemotherapy, serves as a foundational step in this process, effectively reducing the tumor burden and achieving partial or complete remission, thereby setting the stage for a more successful stem cell transplant. Moreover, induction chemotherapy also plays a crucial role in harvesting viable stem cells, essential for the overall treatment efficacy [1, 2].

However, the novel agents used, often a combination of proteasome inhibitors, immunomodulatory drugs, and corticosteroids, carry potential cardiotoxic risks [3]. These cardiotoxic effects can manifest as a spectrum of cardiovascular complications, including heart failure, arrhythmias, hypertension, and ischemic heart disease [4]. Meanwhile, the risk is particularly accentuated in MM patients due to the possibility of preexisting cardiac conditions and age‐related cardiovascular vulnerabilities, and approximately 7.5%–38.1% of patients may experience adverse cardiovascular events [5, 6]. Consequently, the cardiovascular toxicity (CVT) associated with induction chemotherapy adds a layer of complexity to the management of MM and may potentially affect the prognosis of the disease. It is noteworthy that significant cardiovascular structural changes or adverse events in patients undergoing long‐term maintenance chemotherapy typically manifest between Days 200 and 600 [6]. Therefore, it is particularly crucial to early identify covert cardiac remodeling changes related to induction chemotherapy for the timely diagnosis and effective secondary prevention of CVT in MM patients. This is especially important to the small but significant subset of the MM population undergoing the procedure of ASCT with success. In light of this, we observed and analyzed the cardiovascular dynamics in MM patients throughout their induction chemotherapy phase prior to ASCT.

## 2 Methods

### 2.1 Study Population and Protocol

This study enrolled patients with MM who met the selection criteria from January 2020 to December 2023. Our inclusion criteria were as follows: (1) age of ≥ 18 years, (2) currently undergoing or have already completed treatment with anticancer agents at Guangdong Provincial People's Hospital, and (3) provision of written informed consent for data collection. We excluded patients: (1) assessed as ineligible or declined for ASCT, (2) diagnosed with other tumors simultaneously, (3) with concurrent cardiac amyloidosis, (4) absence of cardiac echocardiographic and/or electrocardiographic data prior to and/or following induction chemotherapy, and (5) succumbed or elected for discharge during hospitalization (opting against in‐hospital mortality).

**FIGURE 1:**
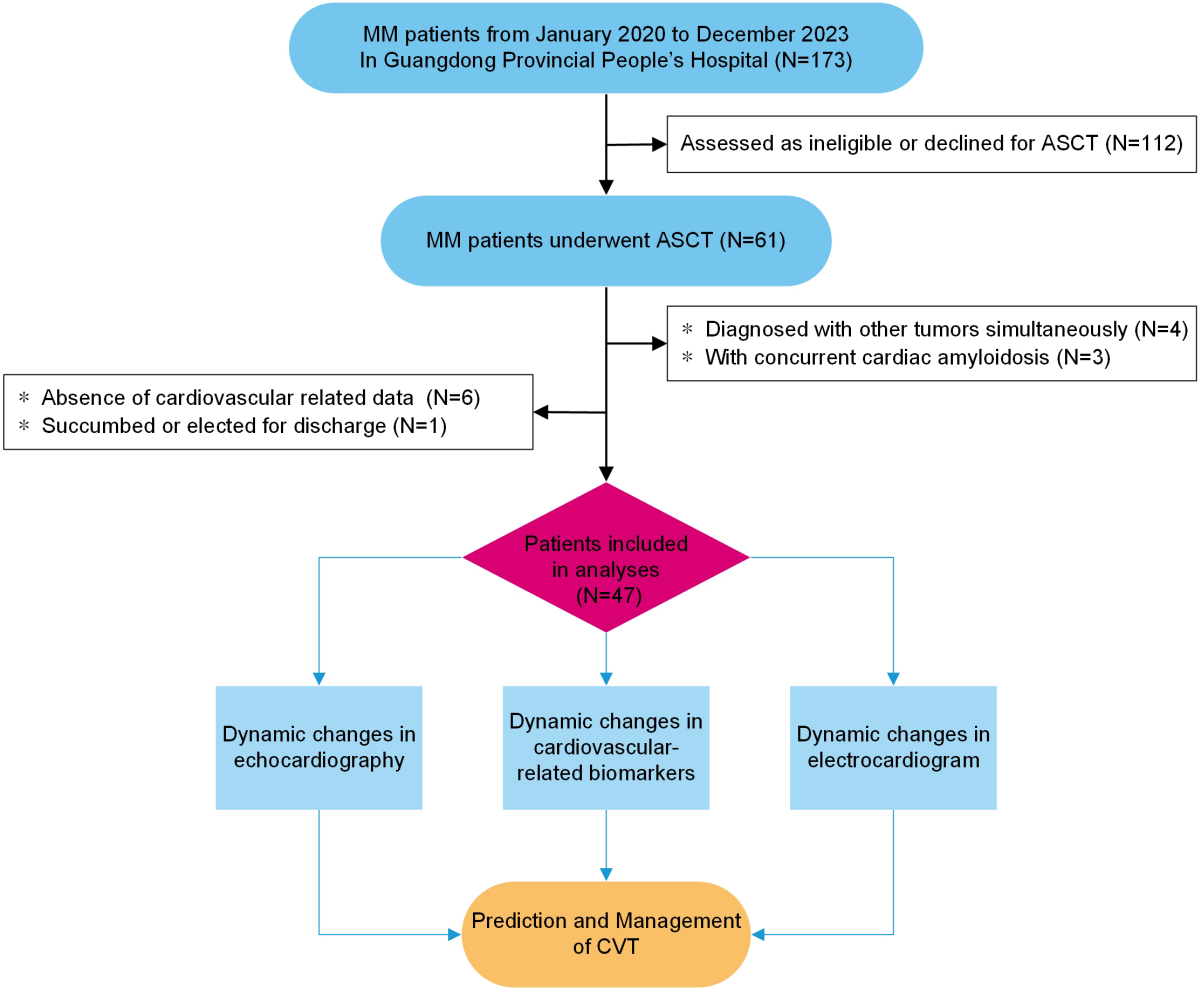
Flowchart of study population and main roadmap. Of the initial 173 patients, 112 were excluded due to ineligibility or declining ASCT, four were excluded for being diagnosed with other simultaneous tumors, three for concurrent cardiac amyloidosis, six for the absence of cardiovascular‐related data, and one for either succumbing to their condition or opting for discharge. Consequently, this left 47 patients who were observed and analyzed for cardiovascular dynamics throughout their induction chemotherapy phase prior to ASCT. The analysis focused mainly on three aspects: structural changes, electrocardiogram alterations, and biomarker levels. ASCT, autologous stem cell transplantation; CVT, cardiovascular toxicity; and MM; multiple myeloma.

Baseline data, including demographic characteristics, comorbidities, laboratory examinations, in‐hospital treatments, and medications prescribed at discharge, were extracted from the electronic clinical management system of the Guangdong Provincial People's Hospital. The baseline assessments were performed at every admission. Comorbidities were determined by the diagnosis before admission or for the first time during hospitalization (Figure 1).

### 2.2 Assessment of Cardiac Dynamic Changes Related to MM Induction Chemotherapy

This study conducts observations and recordings in accordance with the actual treatment timelines used in clinical settings. The assessment of cardiac changes necessitates gathering information from three key perspectives: structural remodeling, electrical remodeling, and biomarker alterations. This process entails collecting baseline data from echocardiograms, electrocardiograms (focused on Lead II), and biomarkers prior to the initiation of induction chemotherapy. Additionally, these examinations are repeated at the end of induction chemotherapy, as well as prior to the commencement of ASCT. The normal value ranges and detection methods for all laboratory test parameters are available in Table S1.

### 2.3 Statistical Analysis

The values were expressed as mean ± standard deviation (SD) or median (interquartile range [IQR]) for continuous variables, according to their distribution. Categorical variables were presented as frequencies (percentages). Comparisons of continuous variables were assessed with independent Student's *t* test, Mann–Whitney *U* test, one‐way analysis of variance (followed by the Dunnett's *t* test for multiple groups), or Kruskal–Wallis *H* test appropriately. Differences in categorical variables were evaluated with *X* ^2^ test or Fisher's exact test (as appropriate). Pearson's correlation test or Spearman's correlation test was used to examine the simple correlation between two continuous variables properly. Paired samples *t* test is employed for pre–post case analysis. Correlation analysis was conducted using binary logistic regression analysis. A two‐tailed *p* < 0.05 was considered as statistical significance. All analyses of data were applied with SPSS version 27 software (SPSS Inc., Chicago, IL, USA).

## 3 Results

### 3.1 Patient Characteristics

Adhering to our inclusion and exclusion criteria, we collected data from 47 patients who underwent comprehensive cardiac analysis (Table 1), including 22 males (46.81%) and 25 females (53.19%), with a mean age of 55.68 ± 11.38 years. These 47 patients received a total of six different induction chemotherapy regimens, including doxorubicin/velcade (bortezomib)/dexamethasone (DVd), ixazomib/revlimid (lenalidomide)/dexamethasone (IRd), ixazomib/thalidomide/dexamethasone (ITd), velcade (bortezomib)/cyclophosphamide/dexamethasone (VCd), velcade (bortezomib)/revlimid (lenalidomide)/dexamethasone (VRd), and combination chemotherapy with treatment periods ranging from 2 to 12 months. The median number of induction chemotherapy cycles was five courses (range: 4–6), with an average duration of 7.10 ± 2.46 months. Response to treatment was categorized based on the Standard International Myeloma Working Group (IMWG) response criteria [7]. Accordingly, 21 patients (44.68%) achieved complete response (CR), 18 patients (38.30%) achieved very good partial response (VGPR), six patients (12.77%) achieved partial response (PR), and two patients (4.26%) achieved minimal response (MR).

**TABLE 1:** Baseline characteristics of MM patients.

Baseline characteristics		All patients (*n* = 47)	Baseline characteristics		All patients (*n* = 47)
Demographics	Female	25 (53.19%)	Chain type	*κ*	23 (48.94%)
	Age (years)	55.68 ± 11.38		*λ*	24 (51.06%)
Blood type	A	9 (19.15%)	Immunophenotyping	IgA	7 (14.89%)
	B	13 (27.66%)		IgD	2 (4.26%)
	AB	3 (6.38%)		IgG	27 (57.45%)
	O	22 (46.81%)		Light chain	9 (19.15%)
Medical history	Smoking	8 (17.02%)		Nonsecretory	2 (4.26%)
	Drinking	5 (10.64%)	Disease staging (R‐ISS)	I	8 (17.02%)
	Hypertension	19 (40.43%)		II	27 (57.45%)
	Hyperlipidemia	13 (27.66%)		III	11 (23.40%)
	Hyperuricemia	10 (21.28%)	Chemotherapy regimen	DVd	1 (2.12%)
	Ischemic heart disease	3 (6.38%)		IRd	4 (8.51%)
	Diabetes	2 (4.26%)		ITd	1 (2.12%)
	Atherosclerosis	4 (8.51%)		VCd	18 (38.30%)
	Chronic kidney disease	22 (46.81%)		VRd	16 (34.04%)
Cardiovascular medication	ACEI/ARBs	2 (4.26%)		VCd + VRd	3 (6.38%)
	Antiplatelet therapy	12 (25.53%)		VCd + ICd	1 (2.12%)
	Anticoagulants	4 (8.51%)		VCd + PVd	1 (2.12%)
	α‐Blockers	1 (2.13%)		VRd + Dara‐Rd	1 (2.12%)
	β‐Blockers	6 (12.77%)		VRd + VPd + Dara‐Pd	1 (2.12%)
	CCBs	7 (14.89%)	Response	CR	21 (44.68%)
	Statins	8 (17.02%)		VGPR	18 (38.30%)
Treatment time (months)		7.10 ± 2.46		PR	6 (12.77%)
Treatment cycle (courses)		5 (4–6)		MR	2 (4.26%)

*Note:* Data are expressed as mean ± SD, number (percentage), or median (interquartile range).

Abbreviations: C, cyclophosphamide; CCBs: calcium channel blockers; CR, complete response; d, dexamethasone; D, doxorubicin; Dara, daratumumab; I, ixazomib; MR, minimal response; P, pomalidomide; PR, partial response; R, revlimid/lenalidomide; T, thalidomide; V, velcade/bortezomib; VGPR, very good partial response.

Among these patients, 65.96% had a history of cardiovascular or endocrine metabolic disorders (including hypertension, hyperlipidemia, hyperuricemia, ischemic heart disease, diabetes, and atherosclerosis) and 46.81% had a history of chronic kidney disease (CKD). However, none of them had a significant history of arrhythmias, heart failure, or hyperthyroidism. Additionally, 59.57% of the patients had a history of taking cardiovascular medications, with 4.26% on ACE inhibitors/ARBs, 12.77% on β‐blockers, and 17.02% on statins.

### 3.2 Alterations in the Primary Disease in MM Before and After Induction Chemotherapy

We conducted a critical evaluation of the primary condition of MM at both the initial and final stages of induction chemotherapy. This assessment involved a brief analysis of the disease's progression, response to the treatment, and changes in key indicators (Table 2). Prior to induction chemotherapy, the levels of serum albumin (34.22 ± 7.58 g/L vs. 39.98 ± 4.66 g/L, 95% CI [−7.71; −3.83], *p* < 0.001), IgM (0.19 [0.07–0.27] g/L vs. 0.33 [0.21–0.51] g/L, *Z* = −4.229, *p* < 0.001), red blood cell (RBC) (2.97 ± 0.79 × 10^12^/L vs. 3.62 ± 0.58 × 10^12^/L, 95% CI [−0.88; −0.43], *p* < 0.001), hemoglobin (HGB) (89.85 ± 24.48 g/L vs. 109.60 ± 17.42 g/L, 95% CI [−26.42; −13.07], *p* < 0.001), hematocrit (HCT) (0.27 ± 0.07 vs. 0.33 ± 0.05, 95% CI [−0.08; −0.04], *p* < 0.001), total cholesterol (TC) (4.22 ± 1.41 mmol/L vs. 5.31 ± 1.33 mmol/L, 95% CI [−1.53; −0.66], *p* < 0.001), high‐density lipoprotein cholesterol (HDL‐C) (1.01 ± 0.33 mmol/L vs. 1.34 ± 0.38 mmol/L, 95% CI [−0.42; −0.23], *p* < 0.001), and low‐density lipoprotein cholesterol (LDL‐C) (2.58 ± 1.02 mmol/L vs. 3.28 ± 0.97 mmol/L, 95% CI [−1.03; −0.36], *p* < 0.001) were lower than their postinduction chemotherapy counterparts. Conversely, the levels of corrected Ca^2+^ (2.43 [2.33–2.61] mmol/L vs. 2.29 [2.21–2.38] mmol/L, *Z* = −4.572, *p* < 0.001), IgG (12.22 [3.99–65.63] g/L vs. 7.98 [5.53–11.62] g/L, *Z* = −3.429, *p* < 0.001), light chain lambda (*λ*) (1.49 [0.44–4.29] g/L vs. 1.02 [0.57–1.59] g/L, *Z* = −2.498, *p* = 0.013), white blood cell (WBC) (6.47 [4.51–8.47] × 10^9^/L vs. 5.17 [3.85–7.56] × 10^9^/L, *Z* = −2.180, *p* = 0.029), lymphocyte proportion (0.28 [0.15%–0.37%] vs. 0.09 [0.03%–0.16%], *Z* = −4.762, *p* < 0.001), and lymphocyte number (1.48 [1.06–2.09] × 10^9^/L vs. 0.51 [0.15–0.82] × 10^9^/L, *Z* = −5.154, *p* < 0.001) were higher before induction chemotherapy compared to after. Furthermore, with regards to kidney function assessment, aside from estimated glomerular filtration rate (eGFR) (85.02 [21.84–102.69] mL/min/1.73m^2^ vs. 94.3 [45.13–111.60] mL/min/1.73m^2^, *Z* = −4.561, *p* < 0.001) which significantly increased following induction chemotherapy, all other indicators significant declined after the induction chemotherapy (*p* < 0.05). However, the statistical analysis revealed no significant variations in the Body Mass Index (BMI), IgA, light chain kappa (*κ*), light chain *κ*/*λ* ratio, hepatic function parameters, triglyceride (TG) levels, blood glucose, and Triglyceride–Glucose (TyG) Index pre‐ and postinduction chemotherapy (*p* > 0.05).

**TABLE 2:** Assessment of the condition of MM patients before and after induction chemotherapy.

	Induction chemotherapy		*p*
	Initial	Final	
BMI (kg/㎡)	21.7 (19.48–25.47)	21.88 (19.82–26.99)	0.064
Serum albumin (g/L)	34.22 ± 7.58	39.98 ± 4.66	< 0.001*
Corrected Ca^2+^ (mmol/L)	2.43 (2.33–2.61)	2.29 (2.21–2.38)	< 0.001*
Serum immunoglobulins			
IgG (g/L)	12.22 (3.99–65.63)	7.98 (5.53–11.62)	**< 0.001** *
IgA (g/L)	0.35 (0.15–1.00)	0.52 (0.19–1.04)	0.961
IgM (g/L)	0.19 (0.07–0.27)	0.33 (0.21–0.51)	**< 0.001** *
Light chain *κ* (g/L)	1.49 (0.68–6.10)	1.95 (1.11–2.74)	0.315
Light chain *λ* (g/L)	1.49 (0.44–4.29)	1.02 (0.57–1.59)	**0.013** *
Light chain *κ*/*λ*	1.25 (0.22–7.72)	1.70 (1.46–2.50)	0.374
Complete blood count (CBC)			
WBC (10^9^/L)	6.47 (4.51–8.47)	5.17 (3.85–7.56)	**0.029** *
RBC (10^12^/L)	2.97 ± 0.79	3.62 ± 0.58	**< 0.001** *
HGB (g/L)	89.85 ± 24.48	109.60 ± 17.42	**< 0.001** *
HCT	0.27 ± 0.07	0.33 ± 0.05	**< 0.001** *
MCV (fL)	92.71 ± 6.30	91.67 ± 6.65	0.162
MCH (pg)	30.20 (28.60–32.10)	30.80 (29.00–32.30)	0.903
MCHC (g/L)	327 (316–337)	332 (320–339)	0.199
PLT (10^9^/L)	197 (145–270)	187 (141–238)	0.077
LYMPHP (%)	0.28 (0.15–0.37)	0.09 (0.03–0.16)	**< 0.001** *
LYMPHN (10^9^/L)	1.48 (1.06–2.09)	0.51 (0.15–0.82)	**< 0.001** *
RDW‐CV	0.15 (0.13–0.17)	0.15 (0.14–0.16)	0.491
RDW‐SD	48.50 (44.70–55.80)	48.90 (46.20–52.70)	0.391
PCT (‰)	1.90 (1.50–2.60)	2.00 (1.50–2.70)	0.701
Renal function (serum)			
β2 Microglobulin (mg/L)	4.91 (2.66–9.94)	2.39 (1.54–4.57)	**< 0.001** *
eGFR (mL/min/1.73m^2^)	85.02 (21.84–102.69)	94.30 (45.13–111.60)	**< 0.001** *
Cr (μmol/L)	86.90 (63.31–233.57)	72.93 (55.31–128.57)	**< 0.001** *
BUN (mmol/L)	6.44 (4.65–14.43)	6.11 (4.66–7.76)	**0.006** *
UA (μmol/L)	456.33 ± 158.77	327.62 ± 114.34	**< 0.001** *
Hepatic function (serum)			
ALT (U/L)	14.00 (9.00–26.00)	14.00 (10.00–21.00)	0.843
AST (U/L)	19.00 (15.00–29.00)	18.00 (15.00–22.00)	0.584
γ‐GT (U/L)	24.00 (16.00–46.00)	23.00 (18.00–31.00)	0.333
ALP (U/L)	85.00 (54.00–118.00)	81.00 (61.00–101.00)	0.377
Glucolipid metabolism levels			
TC (mmol/L)	4.22 ± 1.41	5.31 ± 1.33	**< 0.001** *
TG (mmol/L)	1.33 (1.00–2.04)	1.63 (1.23–2.25)	0.317
HDL‐C (mmol/L)	1.01 ± 0.33	1.34 ± 0.38	**< 0.001** *
LDL‐C (mmol/L)	2.58 ± 1.02	3.28 ± 0.97	**< 0.001** *
Blood glucose (mmol/L)	5.34 (4.62–5.78)	5.23 (4.87–6.52)	0.151
TyG Index	8.61 (8.30–9.22)	8.89 (8.55–9.26)	0.102

*Note:* Data are expressed as mean ± SD or median (interquartile range). Bold and asterisks represent *p*‐values less than 0.05, indicating statistical significance.

Abbreviations: ALP, alkaline phosphatase; ALT, alanine aminotransferase; AST, aspartate transaminase; BMI, Body Mass Index; BUN, blood urea nitrogen; Corrected Ca^2+^, corrected calcium concentration (based on formula: serum total calcium [mmol/L] −0.025 × serum albumin concentration [g/L] + 1.0 [mmol/L]); Cr, creatinine; eGFR, estimated glomerular filtration rate (based on CKD‐EPI 2021 version [8]); HCT, hematocrit; HDL‐C, high‐density lipoprotein cholesterol; HGB, hemoglobin; LDL‐C, low‐density lipoprotein cholesterol; LYMPHN, lymphocyte number; LYMPHP, Lymphocyte Percentage; MCH, mean corpuscular hemoglobin; MCHC, mean corpuscular hemoglobin concentration; MCV, mean corpuscular volume, PCT, plateletcrit; PLT, platelet; RBC, red blood cell; RDW‐CV, coefficient variation of red blood cell volume distribution width; RDW‐SD, standard deviation in red blood cell volume distribution width; TC, total cholesterol; TG, triglyceride; TyG Index, Triglyceride–Glucose Index (based on published formula) [9]: ln (fasting triglycerides [mg/dL] × fasting glucose [mg/dL]/2); UA, uric acid; WBC, white blood cell; γ‐GT, γ‐glutamyl transpeptidase.

* The differences were statistically significant.

### 3.3 Alterations in Cardiac Structural Remodeling Before and After Induction Chemotherapy

Following the administration of induction chemotherapy, there was a statistically significant decrease in several echocardiographic parameters (Table 3), including left ventricular end‐systolic dimension (LVESD) (29.00 [26.00–32.00] mm vs. 28.00 [26.00–31.00] mm, *Z* = −2.383, *p* = 0.017), right atrial diameter (RAD) (44.00 [42.00–48.00] mm vs. 44.00 [41.00–47.00] mm, *Z* = −2.460, *p* = 0.014), E‐wave velocity (0.83 ± 0.24 m/s vs. 0.71 ± 0.23 m/s, 95% CI [0.05; 0.19], *p* = 0.001), E/e' ratio (11.00 [8.00–13.00] vs. 10.00 [8.40–11.40], *Z* = −1.967, *p* = 0.049), and the E/A ratio (1.15 [0.79–1.50] vs. 0.82 [0.68–1.21], *Z* = −4.000, *p* < 0.001), with the latter shifting from a pretreatment value greater than 1 to a posttreatment value less than 1. However, other echocardiographic measures such as left atrial anteroposterior diameter (LAD) and left ventricular ejection fraction (LVEF) did not exhibit statistically significant alterations (*p* > 0.05).

**TABLE 3:** Dynamic changes in echocardiographic, electrocardiographic, and cardiac biomarkers data.

	Induction chemotherapy		*p*	
	Initial	Final		
Blood pressure				
Systolic pressure (mmHg)	126.81 ± 13.73	129.38 ± 15.06	0.280	
Diastolic pressure (mmHg)	77.74 ± 8.86	79.96 ± 10.43	0.197	
Echocardiogram				
AAO (mm)	31.00 (28.00–33.00)	31.00 (29.00–33.00)	0.574	
AO (mm)	28.00 (26.00–30.00)	28.00 (26.00–29.00)	0.731	
MPA (mm)	23.00 (21.00–26.00)	23.00 (21.00–25.00)	0.604	
LAD (mm)	33.00 (31.00–36.00)	33.00 (30.00–36.00)	0.484	
LVEDD (mm)	46.00 (43.00–50.00)	44.00 (41.00–48.00)	0.057	
LVESD (mm)	29.00 (26.00–32.00)	28.00 (26.00–31.00)	**0.017** *	
RAD (mm)	44.00 (42.00–48.00)	44.00 (41.00–47.00)	**0.014** *	
RVD (mm)	50.00 (47.00–54.00)	50.00 (46.00–55.00)	0.524	
IVS (mm)	10.00 (8.00–10.00)	10.00 (9.00–10.00)	0.576	
LVPW (mm)	9.80 (9.00–10.00)	10.00 (9.00–10.00)	0.632	
LVEF (%)	64.49 ± 3.93	64.64 ± 2.81	0.813	
E (m/s)	0.83 ± 0.24	0.71 ± 0.23	**0.001** *	
A (m/s)	0.73 (0.60–0.90)	0.70 (0.64–0.90)	0.079	
E/A ratio	1.15 (0.79–1.50)	0.82 (0.68–1.21)	**< 0.001** *	
E/e' ratio	11.00 (8.00–13.00)	10.00 (8.40–11.40)	**0.049** *	
Electrocardiogram				
HR (bpm)	82.87 ± 12.49	78.77 ± 11.98	**0.020** *	
P‐wave duration (ms)	87.70 ± 15.89	97.85 ± 9.58	**< 0.001** *	
QRS duration (ms)	94.77 ± 11.37	93.26 ± 12.38	0.815	
P‐R duration (ms)	150.00 (133.00–158.00)	152.00 (140.00–168.00)	**0.005** *	
QT interval (ms)	367.87 ± 27.01	377.87 ± 28.48	**0.018** *	
QTc interval	426.30 ± 28.58	427.21 ± 27.61	0.815	
Rv5 (mV)	1.70 ± 0.59	1.59 ± 0.62	0.130	
Sv1 (mV)	0.93 ± 0.57	0.87 ± 0.53	0.180	
Arrhythmic events	3 (6.38%)	9 (19.15%)	—	
Biomarkers of cardiac injury				
ST2 (ng/mL)	14.80 (9.80–31.74)	24.30 (12.50–39.37)	0.065	
NT‐ProBNP (pg/mL)	167.40 (52.50–686.60)	103.10 (40.50–313.50)	**0.009** *	
cTnT (pg/mL)	10.40 (7.10–23.90)	9.90 (6.00–13.50)	**0.008** *	
CK (U/L)	59.00 (34.00–104.00)	51.00 (33.00–79.00)	0.498	
CK‐MB (U/L)	10.00 (10.00–10.00)	10.30 (10.00–13.10)	**0.024** *	
LDH (U/L)	170.00 (134.00–213.00)	177.00 (157.00–206.00)	1.000	
AST (U/L)	19.00 (15.00–29.00)	18.00 (15.00–22.00)	0.584	

*Note:* Bold and asterisks represent *p*‐values less than 0.05, indicating statistical significance.

Abbreviations: AAO, inner diameter of ascending aorta; AO, inner diameter of aorta; AST, aspartate transaminase; CK, creatine kinase; CK‐MB, creatine kinase isoenzyme; cTnT, cardiac troponin T; E&A, peak blood flow velocity of mitral valve orifice; e', early diastolic mitral annular velocity; HR, heart rate; IVS, interventricular septum; LAD, left atrial anteroposterior diameter; LDH, lactate dehydrogenase; LVEDD, left ventricular end‐diastolic dimension; LVEF, left ventricular ejection fraction; LVESD, left ventricular end‐systolic dimension; LVPW, left ventricular posterior wall; MPA, inner diameter of main pulmonary artery; NT‐proBNP, N‐terminal pro‐B‐type natriuretic peptide; QTc, corrected QT interval; RAD, right atrial diameter; RVD, right ventricular basal diameter; ST2, suppression of tumorigenicity 2/growth stimulation expressed gene 2.

* The differences were statistically significant.

Electrocardiographically (Table 3), a significant reduction in heart rate (HR) (82.87 ± 12.49 bpm vs. 78.77 ± 11.98 bpm, 95% CI [0.68; 7.54], *p* = 0.020) was observed, alongside a prolongation of the P‐wave duration (87.70 ± 15.89 mm vs. 97.85 ± 9.58 mm, 95% CI [−15.01; −5.29], *p* < 0.001), P‐R duration (150.00 [133.00–158.00] ms vs. 152.00 [140.00–168.00] ms, *Z* = −2.787, *p* = 0.005), and the QT interval (367.87 ± 27.01 ms vs. 377.87 ± 28.48 ms, 95% CI [−18.20; −1.80], *p* = 0.018). The corrected QT interval (QTc), however, remained statistically unchanged. No significant changes were noted in other electrocardiographic duration or Rv5/Sv1 indices (*p* > 0.05).

In the cohort of MM patients included in the study, none had a documented history of arrhythmias prior to the initiation of induction chemotherapy. However, initial electrocardiographic evaluations identified one case of sinus tachycardia, one instance of Type A Wolff–Parkinson–White (WPW) syndrome, and one patient with incomplete right bundle branch block (RBBB), constituting an overall pretreatment arrhythmia incidence of 6.38%. Following induction chemotherapy, electrocardiograms of the patients with prior sinus tachycardia and Type A WPW syndrome showed no detectable abnormalities. The individual with incomplete RBBB continued to display the same cardiac conduction anomaly, accompanied by a newly developed first‐degree atrioventricular (AV) block. Beyond this case, eight new instances of arrhythmias were identified: one patient developed sinus tachycardia, one exhibited occasional premature ventricular contractions (PVCs), two presented with occasional premature atrial contractions (PACs), one displayed a combination of both PVCs and PACs, and three demonstrated incomplete RBBB. Consequently, the incidence of arrhythmias postinduction chemotherapy escalated to 19.15%, all of which were newly onset conditions.

In terms of cardiac injury biomarkers (Table 3), they have significantly increased compared to the reference values. Of particular note is the slight increase in the level of creatine kinase isoenzyme (CK‐MB) following induction chemotherapy compared to before (10.00 [10.00–10.00] U/L vs. 10.30 [10.00–13.10] U/L, *Z* = −2.260, *p* = 0.024). However, postinduction chemotherapy assessments revealed a significant reduction in the levels of N‐terminal pro‐B‐type natriuretic peptide (NT‐proBNP) (167.40 [52.50–686.60] pg/mL vs. 103.10 [40.50–313.50] pg/mL, *Z* = −2.606, *p* = 0.009) and cardiac troponin T (cTnT) (1040 [7.10–23.90] pg/mL vs. 9.90 [6.00–13.50] pg/mL, *Z* = −2.633, *p* = 0.008), indicating potential mitigation of cardiac injury. Additionally, no significant changes were noted in other suppression of tumorigenicity 2 (ST2), creatine kinase (CK), lactate dehydrogenase (LDH), and AST indices (*p* > 0.05).

### 3.4 Multivariate Analysis of Cardiac Changes Before and After Induction Chemotherapy

In the course of our research into the impacts of induction chemotherapy on cardiac structure, we observed a statistically significant reduction in the cardiac diastolic function parameter, the E/A ratio, which fell below 1 in several instances postinduction chemotherapy. This change, indicating either the emergence or aggravation of cardiac diastolic dysfunction, was found in 24 patients (representing 51.06% of the study population). Through logistic regression analysis focusing on individual variables, we identified correlations between the emergence or aggravation of cardiac diastolic dysfunction and several factors, including the classification type of light chains in MM, the treatment duration of induction chemotherapy, and changes in the blood urea nitrogen levels, heart rate, and the QT interval before and after induction chemotherapy (single‐factor binary logistic regression, *p* < 0.05). However, after adjusting for multiple variables and excluding statistically insignificant factors, only the changes in electrocardiographic QT interval before and after chemotherapy maintained a significant association with the onset or worsening of cardiac diastolic dysfunction, exhibiting odds ratios (OR) of 0.952 (95% CI [0.908–0.999], *p* = 0.046).

In terms of cardiac electrophysiology, our research identified new‐onset arrhythmias in nine patients (representing 19.15% of the study population). Initial logistic regression analyses targeting single variables identified potential associations between the emergence of arrhythmic events and a history of hypertension, use of calcium channel blockers (CCBs), and the classification type of light chains in MM before and after induction chemotherapy. However, integration of these variables into a comprehensive multivariate logistic regression model did not yield statistically significant associations with the emergence of arrhythmias (the meaningful statistical results are presented in Table 4, with additional findings available in the Table S2).

**TABLE 4:** Binary logistic regression analysis to identify adverse cardiac events.

Variables	Emergence or exacerbation of diastolic dysfunction				Emergence of arrhythmic events			
	Univariate analysis		Multivariate analysis		Univariate analysis		Multivariate analysis	
	*p*	OR (95% CI)	*p*	OR (95% CI)	*p*	OR (95% CI)	*p*	OR (95% CI)
Medical History								
Hypertension	0.175	2.286 (0.692–7.554)	—	—	**0.021** *	7.583 (1.366–42.091)	0.293	0.336 (0.044–2.569)
Cardiovascular medication								
CCBs	0.256	2.763 (0.479–15.954)	—	—	**0.013** *	9.333 (1.596–54.578)	0.169	0.214 (0.024–1.925)
Chain type	**0.032** *	3.750 (1.122–12.536)	0.112	3.788 (0.733–19.583)	**0.031** *	11.000 (1.248–96.951)	0.065	8.844 (0.870–89.889)
Treatment time	**0.047** *	1.304 (1.004–1.694)	0.624	1.107 (0.737–1.664)	0.088	1.327 (0.959–1.837)	—	—
Renal function (serum)								
ΔBUN	**0.032** *	0.837 (0.712–0.984)	0.099	0.728 (0.500–1.061)	0.736	0.978 (0.857–1.115)	—	—
Electrocardiogram								
ΔHR	**0.011** *	1.084 (1.019–1.154)	0.458	1.037 (0.941–1.143)	0.484	1.023 (0.960–1.091)	—	—
ΔQT interval	**0.006** *	0.958 (0.930–0.988)	**0.046** *	0.952 (0.908–0.999)	0.189	0.982 (0.956–1.009)	—	—

*Note:* Bold and asterisks represent *p*‐values less than 0.05, indicating statistical significance.

Abbreviations: BUN, blood urea nitrogen; CCBs, calcium channel blockers; HR, heart rate; Δ, the difference in a certain indicator between the initiation and final of induced chemotherapy.

* The differences were statistically significant.

## 4 Discussion

**FIGURE 2:**
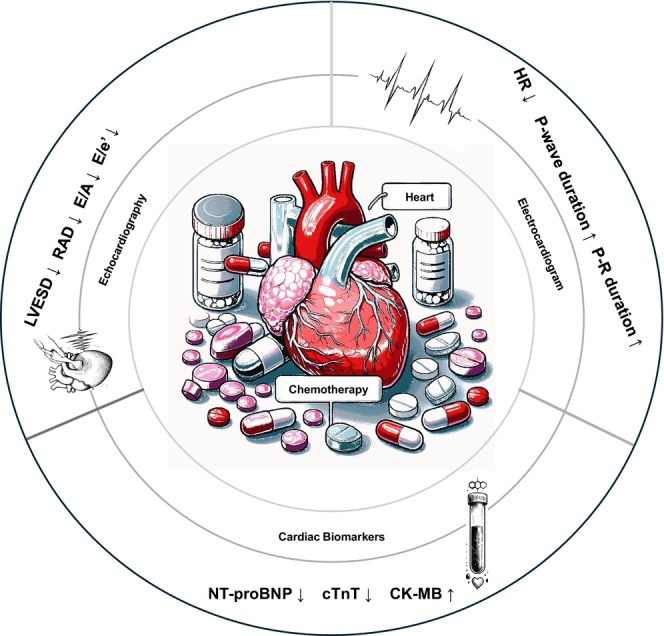
Induction chemotherapy‐related covert cardiac remodeling in preautologous hematopoietic stem cell transplantation for multiple myeloma. CK‐MB, creatine kinase isoenzyme; cTnT, cardiac troponin T; E&A, peak blood flow velocity of mitral valve orifice; e', early diastolic mitral annular velocity; HR, heart rate; LVESD, left ventricular end‐systolic dimension; NT‐proBNP, N‐terminal pro‐B‐type natriuretic peptide; RAD, right atrial diameter.

ASCT is a key treatment for MM patients, involving high‐dose chemotherapy to eliminate cancer cells, followed by transplantation of the patient's own healthy stem cell to rejuvenate bone marrow function. Regardless of age, ASCT extends the median progression‐free survival (PFS) by approximately 11.1 months and improve 5‐year overall survival (OS) by nearly 50%, providing long‐term disease control [10]. Treatment for suitable MM candidates for ASCT involves four stages: pretransplant treatment, induction chemotherapy, transplant, and posttransplant consolidation and maintenance chemotherapy. Recent research indicates that during the early phase of monotherapy maintenance chemotherapy, patients experienced varying degrees of cardiac adverse events, including arrhythmias (2.4%), heart failure (7.3%), ischemic heart disease (1.6%), and sudden cardiac death (0.7%) [5, 11, 12]. The 8‐year overall survival significantly declined to 60% among cancer survivors who developed cardiovascular diseases compared to cancer survivors without cardiovascular diseases [13]. Therefore, timely detection of CVT during the induction chemotherapy phase before ASCT in MM patients is crucial. Given the use of low‐intensity monotherapy in the maintenance chemotherapy stage, which minimizes toxicity, the accumulation of toxic effects typically takes a long time. Hence, these adverse cardiac events are likely associated with the prolonged, multicourse induction chemotherapy in preceding stages, with early indications appearing even before the pretransplant phase but often overlooked due to their subtle and covert nature. This oversight perpetuates cardiotoxic risk during posttransplant consolidation chemotherapy, threatening long‐term survival and treatment outcomes. Nevertheless, there is presently a deficiency in evaluation studies concerning the covert CVT alterations during this phase, a gap that our research endeavors to address. Our findings reveal significant echocardiographic changes indicative of diastolic dysfunction and an increased incidence of arrhythmic events following induction chemotherapy, contributing to the growing body of evidence on the cardiotoxic effects of these treatments (Figure 2).

### 4.1 Echocardiographic Changes and Diastolic Dysfunction

Recent studies by Rhee et al. [14] and Strongman et al. [15] support our findings on echocardiographic changes indicative of heart failure following chemotherapy. However, in contrast to previous research, we have more conclusively discovered that all heart failure events observed in MM patients following induction chemotherapy were cases of heart failure with preserved ejection fraction (HFpEF), characterized primarily by impaired diastolic function, rather than heart failure with reduced ejection fraction (HFrEF), which is more easily identified by noncardiologists through a decreased left ventricular ejection fraction. We found that more than half of MM patients (51.06%) experienced significant worsening of preexisting diastolic dysfunction or newly developed diastolic dysfunction. The decline in E/A ratios and alterations in peak E‐wave velocity observed align with existing evidence, highlighting the cardiotoxicity of induction chemotherapy. These changes, often subclinical, can progress to symptomatic heart failure if unrecognized and unmanaged. Especially, we found that changes in the QT interval before and after induction chemotherapy were significantly associated with the onset or worsening of diastolic dysfunction (OR: 0.952). This suggests that regardless of heart rate, the risk of worsening or new onset of heart failure increases significantly with prolonged QT interval during induction chemotherapy. This reflects the close connection between structural and electrical remodeling and is an important finding that distinguishes this study from others. The mechanisms behind these alterations may involve direct myocardial injury, impaired myocardial relaxation properties, and endothelial dysfunction, further exacerbated by preexisting cardiovascular conditions or the inflammatory milieu of cancer itself. Our observation of declining E/A ratios and altered peak E‐wave velocity are supported by Waxman et al. [16], who summarized similar echocardiographic changes in a larger cohort of MM patients, underscoring the systemic nature of this cardiotoxicity [17]. For example, the incidence of CVT with bortezomib ranges from 2.6% to 5.6%, while secondary heart failure with carfilzomib occurs in 4% to 7% of cases [16]. However, during induction chemotherapy, a combination of multiple drugs is commonly used, with the triplet regimen of proteasome inhibitors (PIs), immunomodulatory drugs (IMiDs), and dexamethasone being the most frequently used in the past [18]. As drug development advances and diverse patient responses are observed in clinical practice, induction chemotherapy regimens in real‐world settings have become increasingly complex. Our study included the induction chemotherapy regimens used in clinical practice prior to ASCT, providing a more accurate reflection of heart failure risk under multiple therapies and offering a basis for disease risk stratification and prediction.

### 4.2 Electrocardiographic Changes and Incidence of Arrhythmic Events

Cardiac arrhythmia is a common complication of chemotherapy, with 17.33% of MM patients experiencing arrhythmic events throughout treatment [19]. Additionally, Tsang et al. [20] reported that 10% of MM patients developed new‐onset atrial fibrillation or atrial flutter after stem cell transplantation. In our research, focused on arrhythmias during the induction chemotherapy phase before ASCT, we found an incidence of 19.15%, primarily manifested as PACs or PVCs, AV block, and incomplete RBBB. Although these arrhythmias may not cause immediate clinical effects, they are early indicators of more severe arrhythmias and warrant further attention. Unfortunately, we did not identify potential predictive factors for arrhythmic events, which may require larger sample sizes and further investigation to elucidate. This discrepancy with studies identifying nonspecific risk factors suggests a multifactorial etiology behind induction chemotherapy‐induced arrhythmogenesis, possibly involving direct electrophysiological disturbances, electrolyte imbalances, or autonomic nervous system disruption. Unlike previous research on chemotherapy‐related arrhythmias, which reported QTc interval prolongation in 12.5% of patients as a hallmark of CVT with various drugs [21], our study found no significant changes in QTc interval during the induction phase. Instead, we observed significant prolongation of P‐wave duration and P‐R interval, signifying distinct alterations in the cardiac conduction system during this phase. These findings underscore the complexity of arrhythmias and their correlation with bradycardia and conduction blocks during induction chemotherapy. Given these findings, we recommend comprehensive cardiac monitoring strategies, including routine electrocardiograms and extended monitoring for high‐risk patients. The identification of arrhythmias warrants a tailored management approach, potentially including chemotherapy adjustments, the use of antiarrhythmic medications, or device therapy in select cases.

### 4.3 Role of Biomarkers in Cardiac Injury Assessment

Previous studies have found that biomarkers such as BNP, TNT, CK, and CK‐MB are associated with chemotherapy‐induced CVT [22]. Both BNP and NT‐proBNP are effective predictors, with elevated levels strongly associated with an increased risk of cardiovascular adverse events. Moreover, patients who experience these events tend to have worse progression‐free and overall survival rates [23, 24]. Intriguingly, our study revealed a distinctive observation in MM patients postinduction chemotherapy. In our cohort study population, despite observing a decline or deterioration in diastolic function, there was no significant disruption in systolic function. However, circulating biomarkers such as NT‐proBNP and cTnT exhibited a notable decrease, a phenomenon previously undocumented in research. This phenomenon could be attributed to two reasons. On one hand, induction chemotherapy reduces tumor burden and improves the primary disease. In MM, overproduction of light chains leads to their deposition in organs, including the heart and kidneys, causing systemic dysfunction. The reduction in serum immunoglobulins, including IgG and light chain λ in our study, likely contributed to the observed improvement in cardiac function. Notably, more than 80% of our patients achieved CR or VGPR, further supporting this observation. This, in turn, may also explain the significant decrease in NT‐proBNP and cTnT levels. On the other hand, the improvement in renal function postinduction chemotherapy is another crucial factor contributing to the decrease in these biomarkers. The heart–kidney interaction, known as cardiorenal syndrome, suggests that dysfunction in one organ can lead to dysfunction in the other. During induction chemotherapy, remission of light‐chain cast nephropathy, a common cause of renal impairment in MM, may lead to a decline in cardiac injury markers associated with renal dysfunction. Renal function evaluations in our MM patients support this notion, demonstrating markedly reduced levels during the initial phases of induction chemotherapy compared to healthy individuals, followed by marked improvement posttreatment. These findings suggest that with a reduction in tumor burden, the multisystem cumulative adverse effects of MM's primary disease are ameliorated. Hence, the clinical significance of these biomarkers may vary across diseases and treatment stages, underscoring the need to interpret them within specific clinical contexts, where their diagnostic, prognostic, and therapeutic implications can differ. In summary, these biomarkers provide a noninvasive method to monitor cardiac function, with changes potentially preceding clinical symptoms or echocardiographic evidence of dysfunction. Incorporating regular cardiac biomarker assessments into the monitoring of MM patients could enable earlier intervention and potentially prevent cardiac injury progression. Further research is needed to establish optimal monitoring intervals and intervention thresholds.

## 5 Conclusion

In conclusion, while induction chemotherapy for MM provides substantial oncologic benefits, it presents covert cardiovascular risks, highlighting the necessity for integrated cardiovascular monitoring and multidisciplinary approach to mitigate these risks.

## Author Contributions

**Chang Dai:** data curation (equal), methodology (equal), writing – original draft (equal). **Weidong Lin:** methodology (equal), writing – original draft (equal). **Fangzhou Liu:** data curation (equal), resources (equal). **Xin Chen:** investigation (equal), methodology (equal). **Yuhan Chen:** investigation (equal), methodology (equal), resources (equal). **Yu Jiang:** investigation (equal), methodology (equal). **Jiaojiao Bai:** investigation (equal), methodology (equal). **Yidong Lv:** formal analysis (equal). **Jianhong Zheng:** investigation (equal), methodology (equal). **Hai Deng:** methodology (equal), resources (equal), validation (equal). **Xin Du:** conceptualization (equal), supervision (equal), writing – review and editing (equal). **Shulin Wu:** conceptualization (equal), methodology (equal), supervision (equal). **Yumei Xue:** conceptualization (equal), supervision (equal), writing – review and editing (equal).

## Ethics Statement

This study was approved by the Guangdong Provincial People's Hospital Ethics Committee (YW2022‐128‐02) and was performed according to the Declaration of Helsinki.

## Consent

Consent was obtained or waived by all participants in this study.

## Conflicts of Interest

The authors declare no conflicts of interest.

## Supporting information


Table S1.
Table S2.

## Data Availability

The data and materials that support the findings of this study are available from the corresponding author (Yumei Xue) upon reasonable request.
