# Supplementary material for: Induction Chemotherapy‐Related Covert Cardiac Remodeling in Pre‐Autologous Hematopoietic Stem Cell Transplantation for Multiple Myeloma: A Retrospective Observational Study
Source: Cancer Med. 2024 Nov 12;13(21):e70329. doi: 10.1002/cam4.70329 (PMC11555718; doi:10.1002/cam4.70329)
Supplement: Supplementary file 1 — Table S1. Table S2. [file CAM4-13-e70329-s001.docx]

**Supplementary materials**

**Table 1** The normal value range and detection method of all laboratory test parameters.

| **Parameters** | **Normal lab range** | | **Method** | |  |
| --- | --- | --- | --- | --- | --- |
| **Serum immunoglobulins** | | | | |  |
| IgG (g/L) | 7.00-16.00 | | Immunonephelometry | |  |
| IgA (g/L) | 0.70-4.00 | | Immunonephelometry | |  |
| IgM (g/L) | 0.40-2.30 | | Immunonephelometry | |  |
| light chain κ (g/L) | 1.38-3.75 | | Immunonephelometry | |  |
| light chain λ (g/L) | 0.93-2.42 | | Immunonephelometry | |  |
| **Complete blood count (CBC)** | | | | |  |
| WBC (10^9^/L) | 3.50-9.50 | Flow Cytometry | | |  |
| RBC (10^12^/L) | 3.80-5.10 | Flow Cytometry | | |  |
| HGB (g/L) | 115-150 | Flow Cytometry | | |  |
| HCT | 0.350-0.450 | Flow Cytometry | | |  |
| MCV (fL) | 82.0-100.0 | Flow Cytometry | | |  |
| MCH (pg) | 27.0-34.0 | Flow Cytometry | | |  |
| MCHC (g/L) | 316-354 | Flow Cytometry | | |  |
| PLT (10^9^/L) | 125-350 | Flow Cytometry | | |  |
| LYMPHP (%) | 0.200-0.500 | Flow Cytometry | | |  |
| LYMPHN (10^9^/L) | 1.10-3.20 | Flow Cytometry | | |  |
| RDW-CV | 0.11-0.16 | Flow Cytometry | | |  |
| RDW-SD | 37.00-54.00 | Flow Cytometry | | |  |
| PCT (‰) | 1.08-2.72 | Flow Cytometry | | |  |
| **Renal function (Serum)** |  |  | |  |  |
| β2 microglobulin (mg/L) | | 0.00-2.80 | Immunonephelometry | | |
| Cr (μmol/L) | 41.00-81.00 | Enzymatic Analysis | | |  |
| BUN (mmol/L) | 3.10-8.80 | Enzymatic Analysis | | |  |
| UA (μmol/L) | 155.0-357.0 | Enzymatic Analysis | | |  |
| **Hepatic function (Serum)** | | | | |  |
| ALT (U/L) | 9-50 | Rate Method | | |  |
| AST (U/L) | 13-35 | Rate Method | | |  |
| γ-GT (U/L) | 7-45 | Rate Method | | |  |
| ALP (U/L) | 50-135 | Rate Method | | |  |
| **Glucolipid metabolism levels** | | | | |  |
| TC (mmol/L) | 0.00-6.22 | Enzymatic Analysis | | |  |
| TG (mmol/L) | 0.00-1.70 | Enzymatic Analysis | | |  |
| HDL-C (mmol/L) | 1.29-1.55 | Enzymatic Analysis | | |  |
| LDL-C (mmol/L) | 0.00-4.14 | Enzymatic Analysis | | |  |
| Blood glucose (mmol/L) | 3.90-6.10 | Enzymatic Analysis | | |  |
| **Biomarkers of cardiac injury** | | | | |  |
| ST2 (ng/mL) | 0.00-35.00 | Microfluidic immunofluorescence method | | |  |
| NT-ProBNP (pg/mL) | 0.0-125.0 | Electro-chemiluminescence Immunoassay | | |  |
| cTnT (pg/mL) | 0.0-14.0 | Electro-chemiluminescence Immunoassay | | |  |
| CK (U/L) | 50-310 | Rate Method | | |  |
| CK-MB (U/L) | 0-25 | Rate Method | | |  |
| LDH (U/L) | 120-250 | Rate Method | | |  |
| AST (U/L) | 13-35 | Rate Method | | |  |

**Abbreviations:** WBC: White blood cell, RBC: Red blood cell, HGB: Hemoglobin, HCT: Hematocrit, MCV: Mean corpusular volume, MCH: Mean corpusular hemoglobin, MCHC: Mean corpusular hemoglobin concentration, PLT: Platelet, LYMPHP: Lymphocyte Percentage, LYMPHN: Lymphocyte Number, RDW-CV: Coefficient variation of red blood cell volume distribution width, RDW-SD: Standard deviation in red blood cell volume distribution width, PCT: Plateletocrit, Cr: Creatinine, BUN: Blood urea nitrogen, UA: Uric acid, ALT: Alanine aminotransferase, AST: Aspartate transaminase, γ-GT: γ-glutamyl transpeptidase, ALP: Alkaline phosphatase, TC: Total cholesterol, TG: Triglyceride, HDL-C: High-density lipoprotein cholesterol, LDL-C: Low-density lipoprotein cholesterol, ST2: Suppression of tumorigenicity 2/ Growth stimulation expressed gene 2, NT-proBNP: N terminal pro B type natriuretic peptide, cTnT: cardiac troponin T, CK: Creatine kinase, CK-MB: Creatine kinase isoenzyme, LDH: Lactate dehydrogenase.

**Table 2** Binary logistic regression analysis to identify adverse cardiac events.

| **Variables** | **Emergence or exacerbation of diastolic dysfunction** | | | | **Emergence of arrhythmic events** | | | |
| --- | --- | --- | --- | --- | --- | --- | --- | --- |
|  | **Univariate analysis** | | **Multivariate analysis** | | **Univariate analysis** | | **Multivariate analysis** | |
|  | ***P*-value** | **OR (95% CI)** | ***P*-value** | **OR (95% CI)** | ***P*-value** | **OR (95% CI)** | ***P*-value** | **OR (95% CI)** |
| Age | 0.112 | 1.045 (0.990-1.104) | - | - | 0.721 | 1.012 (0.948-1.081) | - | - |
| Male gender | 0.109 | 0.381 (0.117-1.241) | - | - | 0.116 | 3.889 (0.714-21.194) | - | - |
| **Medical History** | | | | | | | | |
| Smoking | 0.405 | 0.514 (0.108-2.456) | - | - | 0.604 | 0.554 (0.059-5.173) | - | - |
| Drinking | 0.674 | 1.500 (0.227-9.916) | - | - | 0.959 | 1.062 (0.104-10.841) | - | - |
| Hypertension | 0.175 | 2.286 (0.692-7.554) | - | - | **0.021** | 7.583 (1.366-42.091) | 0.293 | 0.336 (0.044-2.569) |
| Hyperlipidemia | 0.814 | 1.167 (0.324-4.200) | - | - | 0.673 | 1.400 (0.293-6.680) | - | - |
| Hyperuricemia | 0.940 | 0.947 (0.234-3.831) | - | - | 0.939 | 1.071 (0.185-6.193) | - | - |
| Ischaemic heart disease | 0.534 | 0.457 (0.039-5.409) | - | - | 0.528 | 2.250 (0 .181-27.957) | - | - |
| Diabetes | 0.975 | 0.957 (0.056-16.254) | - | - | 0.297 | 4.625 (0.261-81.999) | - | - |
| Atherosclerosis | 0.965 | 0.955 (0.123-7.408) | - | - | 0.130 | 5.143 (0.617-42.872) | - | - |
| Chronic kidney disease | 0.304 | 1.838 (0.576-5.865) | - | - | 0.560 | 1.544 (0.358-6.662) | - | - |
| **Cardiovascular medication** | | | | | | | | |
| ACEI/ARBs | 0.999 | 0.957 (0.882-17.526) | - | - | 0.297 | 4.625 (0.261-81.999) | - | - |
| Antiplatelet | 0.453 | 0.602 (0.160-2.266) | - | - | 0.159 | 3.000 (0.650-13.836) | - | - |
| Anticoagulants | 0.965 | 0.955 (0.123-7.408) | - | - | 0.999 | 0.000 (0.000-0.000) | - | - |
| α-Blockers | 1.000 | 0.000 (0.000-0.000) | - | - | 1.000 | 0.000 (0.000-0.000) | - | - |
| β-Blockers | 0.420 | 2.100 (0.346-12.761) | - | - | 0.356 | 2.429 (0.371-5.951) | - | - |
| CCBs | 0.256 | 2.763 (0.479-15.954) | - | - | **0.013** | 9.333 (1.596-54.578) | 0.169 | 0.214 (0.024-1.925) |
| Statins | 0.405 | 1.944 (0.407-9.287) | - | - | 0.646 | 0.656 (0.109-3.959) | - | - |
| Chain type | **0.032** | 3.750 (1.122-12.536) | 0.112 | 3.788 (0.733-19.583) | **0.031** | 11.000 (1.248-96.951) | 0.065 | 8.844 (0.870-89.889) |
| Immunophenotyping | 0.999 | 0.000 (0.000-0.000) | - | - | 0.999 | 0.000 (0.000-0.000) | - | - |
| Disease Staging (R-ISS) | 0.472 | 0.714 (0.285-1.788) | - | - | 0.810 | 1.150 (0.367-3.609) | - | - |
| Chemotherapy regimen | 1.000 | 1.000 (0.000-0.000) | - | - | 0.999 | 0.000 (0.000-0.000) | - | - |
| Treatment Time | **0.047** | 1.304 (1.004-1.694) | 0.624 | 1.107 (0.737-1.664) | 0.088 | 1.327 (0.959-1.837) | - | - |
| Treatment cycle | 0.102 | 1.414 (0.934-2.140) | - | - | 0.169 | 1.374 (0.874-2.159) | - | - |
| **Blood test** | | | | | | | | |
| Serum albumin | 0.402 | 1.055 (0.930-1.198) | - | - | 0.733 | 0.973 (0.833-1.137) | - | - |
| ΔSerum albumin | 0.938 | 0.997 (0.913-1.088) | - | - | 0.491 | 0.959 (0.853-1.080) | - | - |
| Corrected Ca^2+^ | 0.219 | 0.040 (0.000-6.790) | - | - | 0.320 | 27.764 (0.040-1.941×10^4^) | - | - |
| ΔCorrected Ca^2+^ | 0.212 | 3.465 (0.491-24.444) | - | - | 0.588 | 2.025 (0.158-26.003) | - | - |
| **Serum immunoglobulins** | | | | | | | | |
| IgG | 0.455 | 0.968 (0.889-1.054) | - | - | 0.315 | 0.900 (0.732-1.106) | - | - |
| ΔIgG | 0.863 | 1.002 (0.984-1.019) | - | - | 0.417 | 1.010 (0.986-1.035) | - | - |
| IgM | 0.445 | 0.458 (0.062-3.399) | - | - | 0.456 | 0.330 (0.018-6.107) | - | - |
| ΔIgM | 0.980 | 0.976 (0.154-6.195) | - | - | 0.184 | 0.137 (0.007-2.582) |  |  |
| light chain λ | 0.491 | 0.898 (0.661-1.220) | - | - | 0.432 | 0.650 (0.222-1.905) | - | - |
| Δlight chain λ | 0.107 | 0.854 (0.706-1.034) | - | - | 0.919 | 0.992 (0.851-1.156) | - | - |
| **Complete blood count (CBC)** | | | | | | | | |
| WBC | 0.846 | 1.006 (0.950-1.064) | - | - | 0.779 | 0.988 (0.907-1.076) | - | - |
| ΔWBC | 0.971 | 1.001 (0.945-1.061) | - | - | 0.668 | 0.978 (0.883-1.083) | - | - |
| RBC | 0.450 | 1.480 (0.535-4.097) | - | - | 0.356 | 1.865 (0.497-7.000) | - | - |
| ΔRBC | 0.948 | 0.975 (0.453-2.097) | - | - | 0.588 | 1.315 (0.489-3.533) | - | - |
| HGB | 0.701 | 1.007 (0.974-1.041) | - | - | 0.583 | 1.012 (0.969-1.057) | - | - |
| ΔHGB | 0.970 | 1.000 (0.974-1.025) | - | - | 0.642 | 1.008 (0.976-1.040) | - | - |
| HCT | 0.653 | 15.813 (0.000-2.682×10^6^) | - | - | 0.504 | 231.026 (0.000-1.986×10^9^) | - | - |
| ΔHCT | 0.999 | 1.003 (0.000-6317.081) | - | - | 0.675 | 11.136 (0.000-8.742×10^5^) | - | - |
| LYMPHP | 0.674 | 0.424 (0.008-23.121) | - | - | 0.125 | 0.000 (0.000-14.775) | - | - |
| ΔLYMPHP | 0.342 | 0.238 (0.012-4.602) | - | - | 0.285 | 0.103 (0.002-6.660) | - | - |
| LYMPHN | 0.643 | 1.192 (0.567-2.507) | - | - | 0.455 | 0.581 (0.140-2.415) | - | - |
| ΔLYMPHN | 0.444 | 0.774 (0.402-1.490) | - | - | 0.121 | 0.462 (0.174-1.226) | - | - |
| **Renal function (Serum)** | | | | | | | | |
| β2 microglobulin | 0.515 | 1.023 (0.955-1.096) | - | - | 0.538 | 0.943 (0.782-1.136) | - | - |
| Δβ2 microglobulin | 0.461 | 0.976 (0.916-1.041) | - | - | 0.403 | 1.060 (0.924-1.217) | - | - |
| eGFR | 0.153 | 0.988 (0.971-1.005) | - | - | 0.820 | 1.002 (0.982-1.023) | - | - |
| ΔeGFR | 0.709 | 0.994 (0.962-1.027) | - | - | 0.300 | 0.971 (0.920-1.026) | - | - |
| Cr | 0.821 | 1.000 (0.997-1.002) | - | - | 0.470 | 0.995 (0.981-1.009) | - | - |
| ΔCr | 0.115 | 0.995 (0.990-1.001) | - | - | 0.500 | 1.002 (0.996-1.009) | - | - |
| BUN | 0.391 | 0.954 (0.858-1.062) | - | - | 0.475 | 0.924 (0.743-1.148) | - | - |
| ΔBUN | **0.032** | 0.837 (0.712-0.984) | 0.099 | 0.728 (0.500-1.061) | 0.736 | 0.978 (0.857-1.115) | - | - |
| UA | 0.089 | 1.005 (0.999-1.010) | - | - | 0.805 | 0.999 (0.993-1.006) | - | - |
| ΔUA | 0.427 | 0.998 (0.994-1.003) | - | - | 0.821 | 1.001 (0.995-1.006) | - | - |
| **Lipid metabolism** | | | | | | | | |
| TC | 0.616 | 1.118 (0.722-1.731) | - | - | 0.467 | 0.812 (0.464-1.422) | - | - |
| ΔTC | 0.238 | 1.276 (0.851-1.912) | - | - | 0.528 | 0.852 (0.517-1.402) | - | - |
| HDL-C | 0.546 | 0.620 (0.131-2.924) | - | - | 0.271 | 2.934 (0.432-19.918) | - | - |
| ΔHDL-C | 0.695 | 1.435 (0.235-8.748) | - | - | 0.858 | 1.234 (0.125-12.197) | - | - |
| LDL-C | 0.532 | 1.211 (0.665-2.205) | - | - | 0.341 | 0.688 (0.319-1.486) | - | - |
| ΔLDL-C | 0.194 | 1.428 (0.834-2.446) | - | - | 0.487 | 0.793 (0.412-1.525) | - | - |
| **Echocardiogram** | | | | | | | | |
| LVESD | 0.471 | 1.056 (0.911-1.224) | - | - | 0.959 | 1.005 (0.837-1.207) | - | - |
| ΔLVESD | 0.466 | 0.954 (0.839-1.083) | - | - | 0.359 | 1.081 (0.915-1.277) | - | - |
| RAD | 0.375 | 1.051 (0.942-1.173) | - | - | 0.983 | 0.999 (0.875-1.139) | - | - |
| ΔRAD | 0.098 | 0.899 (0.793-1.020) | - | - | 0.177 | 0.897 (0.765-1.051) |  |  |
| E | Indicators for assessing the outcome. | | | | 0.871 | 0.766 (0.031-19.198) | - | - |
| ΔE | Indicators for assessing the outcome. | | | | 0.323 | 0.225 (0.012-4.324) | - | - |
| E/A | Indicators for assessing the outcome. | | | | 0.159 | 0.128 (0.007-2.232) | - | - |
| ΔE/A | Indicators for assessing the outcome. | | | | 0.464 | 0.479 (0.067-3.436) | - | - |
| E/e’ | 0.626 | 1.036 (0.898-1.195) | - | - | 0.054 | 1.185 (0.997-1.408) | - | - |
| ΔE/e’ | 0.314 | 0.919 (0.780-1.083) | - | - | 0.553 | 0.944 (0.782-1.141) | - | - |
| **Electrocardiogram** | | | | | | | | |
| HR | 0.531 | 1.016 (0.967-1.067) | - | - | 0.512 | 1.020 (0.962-1.081) | - | - |
| ΔHR | **0.011** | 1.084 (1.019-1.154) | 0.458 | 1.037 (0.941-1.143) | 0.484 | 1.023 (0.960-1.091) | - | - |
| P-wave duration | 0.179 | 1.045 (0.980-1.114) | - | - | 0.242 | 1.050 (0.968-1.140) | - | - |
| ΔP-wave duration | 0.153 | 1.031 (0.989-1.074) | - | - | 0.356 | 1.020 (0.978-1.063) | - | - |
| P-R duration | 0.070 | 1.028 (0.998-1.060) | - | - | 0.289 | 1.017 (0.986-1.049) | - | - |
| ΔP-R duration | 0.376 | 1.017 (0.979-1.057) |  |  | 0.729 | 0.992 (0.945-1.040) |  |  |
| QT interval | 0.155 | 0.985 (0.964-1.006) | - | - | 0.572 | 1.007 (0.982-1.034) | - | - |
| ΔQT interval | **0.006** | 0.958 (0.930-0.988) | **0.046** | 0.952 (0.908-0.999) | 0.189 | 0.982 (0.956-1.009) | - | - |
| QTc interval | 0.138 | 0.982 (0.959-1.006) | - | - | 0.159 | 1.020 (0.992-1.047) | - | - |
| ΔQTc interval | 0.071 | 0.977 (0.953-1.002) | - | - | 0.207 | 0.982 (0.955-1.010) | - | - |
| **Biomarkers of cardiac injury** | | | | | | | | |
| NT-ProBNP | 0.545 | 1.000 (0.999-1.000) | - | - | 0.728 | 1.000 (0.999-1.001) | - | - |
| ΔNT-ProBNP | 0.571 | 1.000 (0.999-1.000) | - | - | 0.474 | 1.000 (0.999-1.002) | - | - |
| cTnT | 0.926 | 1.003 (0.949-1.059) | - | - | 0.896 | 1.005 (0.939-1.075) | - | - |
| ΔcTnT | 0.119 | 0.957 (0.906-1.011) | - | - | 0.250 | 1.045 (0.970-1.126) | - | - |
| CK-MB | 0.925 | 1.006 (0.886-1.143) | - | - | 0.471 | 1.054 (0.913-1.218) | - | - |
| ΔCK-MB | 0.316 | 1.047 (0.957-1.145) | - | - | 0.310 | 1.069 (0.940-1.216) | - | - |

**Abbreviations**: CCBs: Calcium channel blockers, Corrected Ca2+: Corrected calcium concentration (based on formula, serum total calcium[mmol/L] - 0.025 × serum albumin concentration [g/L] + 1.0 [mmol/L]), WBC: White blood cell, RBC: Red blood cell, HGB: Hemoglobin, HCT: Hematocrit, LYMPHP: Lymphocyte Percentage, LYMPHN: Lymphocyte Number, eGFR: Estimated glomerular filtration rate (based on CKD-EPI 2021 version[7]), Cr: Creatinine, BUN: Blood urea nitrogen, UA: Uric acid, TC: Total cholesterol, HDL-C: High-density lipoprotein cholesterol, LDL-C: Low-density lipoprotein cholesterol, LVESD: Left ventricular end-systolic dimension, RAD: Right atrial diameter, E&A: Peak blood flow velocity of mitral valve orifice, e’: Early diastolic mitral annular velocity, HR: Heart rate, QTc: corrected QT interval, NT-proBNP: N terminal pro B type natriuretic peptide, cTnT: cardiac troponin T, CK-MB: Creatine kinase isoenzyme.

*The differences were statistically significant.
